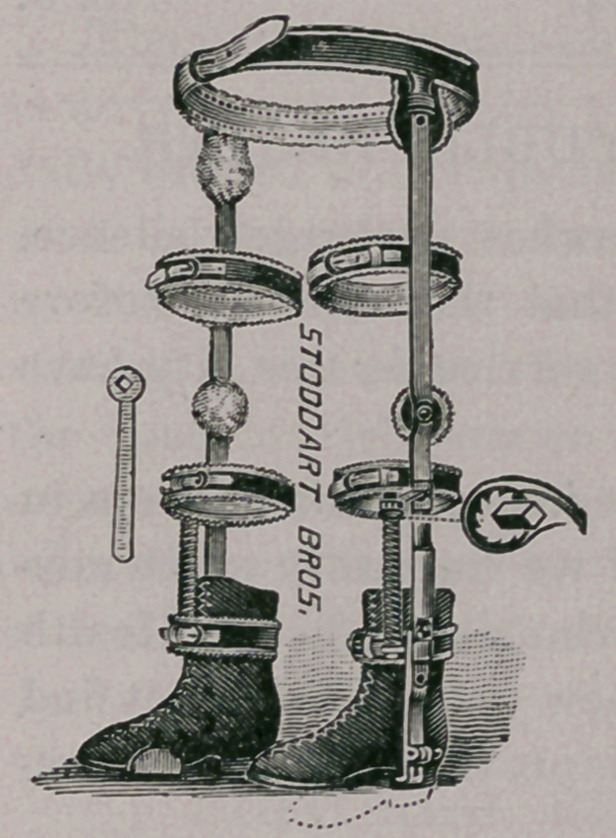# A New and Improved Form of Club-Foot Shoe

**Published:** 1892-01

**Authors:** Roswell Park

**Affiliations:** Buffalo, N. Y.


### A NEW AND IMPROVED FORM OF CLUB-FOOT SHOE.

By ROSWELL PARK, M. D., Buffalo, N. Y.

THE ideal club-foot shoe, which shall be both *light*, *durable*, and *effective* in the highest degree, has, perhaps, not yet been constructed. That form, however, which is represented in the accompanying illustration, more nearly attains to my ideal than any that I have yet used. It is practically a combination of the good features of several others, and as made for me by Messrs. Stoddart Bros.' excellent workmen, it accomplishes its purpose admirably.

Like all surgeons, I have found it necessary in the majority of cases of club-feet to correct not only the displacement of the tarsal bones and their distortion, but to overcome an inward (or outward) rotation of the tibias, sometimes even of the femurs. By a swivel-joint a little above the ankle, and, if necessary, by another above the knee, operated by a spiral spring with ratchet-joint, any desired amount of internal or external rotary tension or twist can be given. It is intended that this apparatus shall be worn night and day, at least for a time, in order that an impetus to growth in the corrected position may thus be given. Of course, for this purpose, the apparatus must be connected with a pelvic band. *All other forms* not thus giving a firm point of support *fail to accomplish their purpose.*

By means of one or two lock-joints about the ankle or near the sole, any desired position of the foot may be secured and maintained.

For two years I have used this apparatus, and a considerable number of cases has served to test its merits and efficiency. Thus far it has proved to be the least disappointing and the most reliable of any that I have ever used. There are no wire chains or rubber cords about it, nor any hooks or eyes nor other parts to break off easily, and if properly constructed it will be found to far outlast the common shoes made and sold for the purpose.

## Figures and Tables

**Figure f1:**